# Bilateral peripheric facial nerve palsy following SARS‐CoV‐2 infection: A case report and review of literature

**DOI:** 10.1002/ccr3.7034

**Published:** 2023-03-02

**Authors:** Majid Dezfouli, Firouzeh Moeinzadeh, Farzin Ghiasi, Neda Abdeyazdan, Sadegh Mazaheri‐Tehrani

**Affiliations:** ^1^ Department of Internal Medicine Isfahan University of Medical Sciences Isfahan Iran; ^2^ Isfahan Kidney Diseases Research Center Isfahan University of Medical Sciences Isfahan Iran; ^3^ Department of Internal medicine, Division of Pulmonary Disease, School of Medicine Isfahan University of Medical Sciences Isfahan Iran; ^4^ Student Research Committee, School of Medicine Isfahan University of Medical Sciences Isfahan Iran

**Keywords:** bell palsy, bilateral facial palsy, COVID‐19, facial paralysis, SARS‐CoV‐2

## Abstract

Neurologic complications of SARS‐CoV‐2 infection have been reported commonly. Peripheric facial nerve palsy is one of the most reported neurologic problems. However, idiopathic bilateral facial palsy is a very rare complication of SARS‐CoV‐2 infection. Herein, we present a case of a COVID‐19 35‐year‐old man, which developed bilateral facial palsy.

## Abstract 2

Idiopathic bilateral facial palsy (also known as bilateral bell's palsy) is a very rare complication of SARS‐CoV‐2 infection. It has some important differential diagnosis that should rule out.

## 1 INTRODUCTION

The novel coronavirus disease (COVID‐19) has dramatically affected public health since December 2019. Although the catastrophic days of the pandemic are over, COVID‐19 is still considered a significant health crisis.^1^ Various complications of COVID‐19 besides respiratory symptoms have been reported, including cardiovascular,^2^ hematological,^3^ rheumatological,^4^ and neurological.^5^ A study in China reported neurologic complications in about 36.4% of COVID‐19 patients.^5^ Facial palsy is one of the major neurologic findings in COVID‐19 patients. There are many cases of unilateral facial palsy due to COVID‐19,^6^ but to the best of our knowledge, there are few cases with idiopathic bilateral facial palsy (BFP) as a complication of COVID‐19.^7^, ^8^, ^9^, ^10^

The condition known as Bell's palsy is a type of facial nerve palsy that can be idiopathic or of suspected viral etiology. However, herpes simplex virus (HSV) activation is the likely cause of Bell's palsy in most cases, although there is no well‐established, widely available method of confirming a viral mechanism in clinical practice. Inflammation starts at the geniculate ganglion, extending proximally and distally, causing facial palsy due to neural edema inside the narrow facial canal. Laboratory tests are usually not indicated. There are several differential diagnoses, including Lyme disease, neoplasia, Ramsay Hunt Syndrome, and sarcoidosis.^11^ A detailed history‐taking usually facilitates the diagnosis. The magnetic resonance imaging (MRI) findings are nonspecific and typically include facial nerve enhancement.^12^

Herein, we present a case of isolated BFP due to COVID‐19 without other neurologic complications or any underlying diseases.

## 2 CASE PRESENTATION

A 35‐year‐old man was admitted with a chief complaint of fever and loss of consciousness. He had a history of acute watery diarrhea 5 days before admission, and since last night he had developed a high‐grade fever (105.8 °F) and a significant decrease in his consciousness. He had no history of a similar condition and any underlying diseases. His family mentioned a history of contact with COVID‐19 patients (his brother and mother) for about a week. He had injected his last dose of Sinopharm COVID‐19 vaccine (second dose) 8 months ago. He had no history of allergy to any specific drug or food and even no history of trauma.

On admission, his blood pressure was 100/60 mmHg, pulse rate was 90/min, respiratory rate was 18/min, the temperature was 98.6 °F, and O2 saturation was 95%. He was not conscious, but he withdrew from pain, and open his eyes. His pupils were reactive and normal size, and the gag reflex was normal. The Glasgow Coma Scale (GSC) was 10/15 at the time of admission (E: 3, M: 4, V: 3). Bilateral bronchial breathing sounds and the abdominal examination were normal. There was no other noteworthy finding in the physical examination. His laboratory results on admission were serum Na, 117 mEq/L; K, 3.1 mEq/L; BUN, 11 mg/dL; creatinine 0.9 mg/dL; INR, 1.3; hemoglobin, 13.8 g/dL; WBC, 6900; platelets, 132,000. On the other hand, his SARS‐CoV‐2 PCR was positive, but he had no respiratory signs, and his chest high‐resolution computed tomography (HRCT) was normal.

**FIGURE 1:**
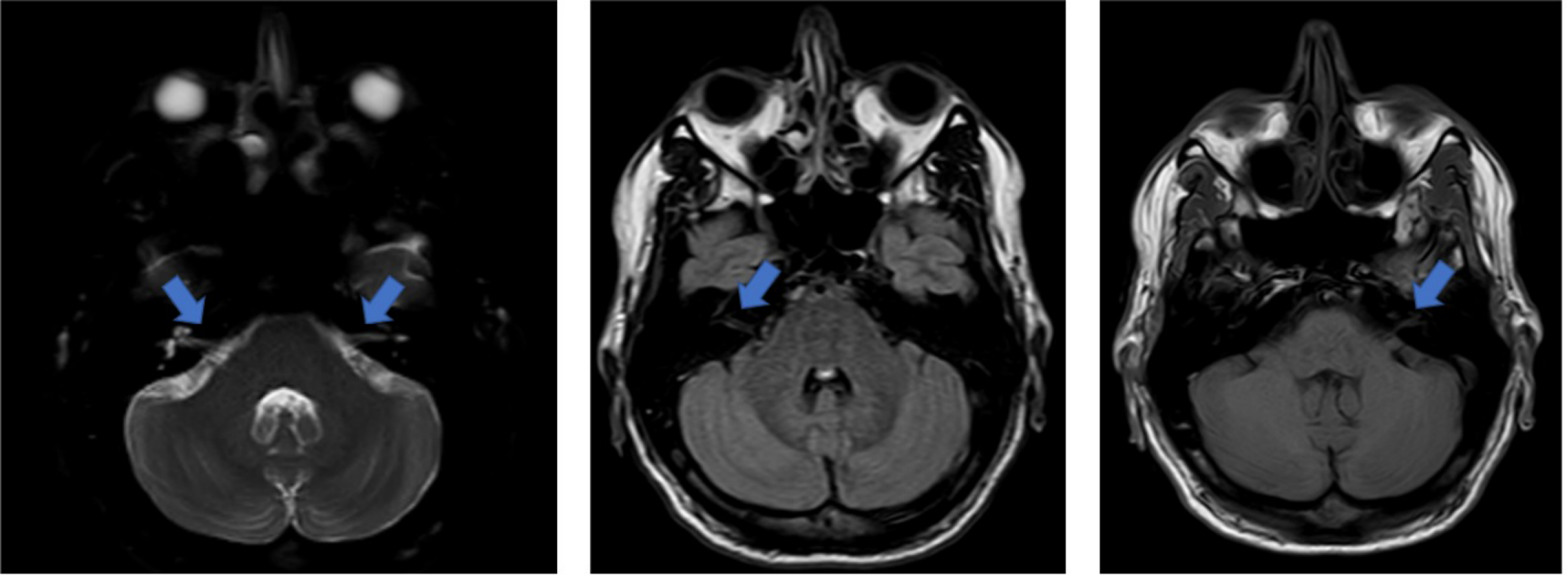
Normal VII‐VIII nerves in brain MRI.

His consciousness improved after an increase in the serum Na level (137 mEq/L). On the following day, although his blood test indicated normal metabolic and electrolyte profiles and his consciousness has improved (GCS: 13/15, E: 3, M: 6, V: 4), in the neurologic examination, a bilateral facial nerve weakness, reduced eyelid closure, drooling from both sides of the mouth, and slurred speech was detected, moreover, he could not raise his eyebrows, puff his cheeks, and keep food in his mouth (House‐Brackmann grade V on both sides), thus a nasogastric tube was inserted for him. The rest of the neurologic examinations including other cranial nerves, deep tendon reflexes, sensory examinations, and gait were normal. He moved his lower and upper limbs symmetrically, and there was no sign of focal neurologic deficit and his lower and upper limbs muscle strength test was normal. The brain MRI and brain CT (1 day after BFP onset) were normal, but unfortunately, the patient refused to undergo MRI with contrast. VII‐VIII nerves are demonstrated in Figure 1. Lumbar puncture (LP) showed normal glucose (67 mg/dL), elevated protein levels (69 mg/dL), and no WBCs, RBC, or any bacterial or viral contamination.

According to the normal neurologic examinations (normal deep tendon reflexes, normal sensory examinations, and normal power of the limbs) on one hand, and no progression in the neurologic disorders during the hospitalization period, on the other hand, the possibility of Guillain–Barre syndrome (GBS) is low. However, our patient refused to undergo electromyography, in order to definitely reject the possibility of GBS. The serology tests for different possible infections such as human immunodeficiency virus (HIV), Epstein–Barr virus (EBV), syphilis, HSV, and cytomegalovirus were negative. There was no sign of neoplasm, local infections, or any other causes of BFP in the ear–nose–throat examination. His rheumatological tests including antinuclear antibody (ANA), anti‐neutrophil cytoplasmic antibodies (ANCA), and complement components 3 and 4 (C3 and C4) were negative. Thus, all the possible causes had been ruled out, and our final diagnosis was idiopathic BFP (Bell's palsy) due to severe acute respiratory syndrome coronavirus 2 (SARS‐CoV‐2) infection.

Before LP was done, due to the probability of encephalitis and meningitis, corticosteroids (Prednisolone 50 mg per day for 1 week), Remdesivir, Acyclovir, Vancomycin, and Ceftriaxone were initiated. But after the negative result of LP for HSV, SARS‐CoV‐2, and bacterial contamination, we discontinue mentioned drugs and taper the corticosteroid course with Prednisolone 10 mg per day. His facial nerve examination improved during hospitalization. The nasogastric tube was removed after 2 weeks, and he could swallow with no problem. He was discharged after 21 days of BFP initiation, with normal facial nerve function (House‐Brackmann grade I).

## 3 DISCUSSION

The exact pathogenesis of facial palsy due to COVID‐19 has remained unclear, but the angiotensin‐converting enzyme 2 (ACE2) receptor entranceway is the most probable mechanism. ACE2 receptors are widely expressed in various tissues, especially neurons, which make them at risk of SARS‐CoV‐2 invasion.^13^ The pathophysiology of peripheric facial nerve palsy may also be related to increased viral replication and dissemination in the axon, leading to demyelination and inflammation.^14^ Other, suggested mechanisms are accessing cranial nerves through blood circulation, or retrogradely transferring into the nervous system through olfactory nerves.^13^ In our case, due to negative SARS‐CoV‐2 PCR in cerebrospinal fluid (CSF), immune‐mediated pathways might account for BFP.

BFP is an extremely rare condition. About 0.3% of patients with facial palsy, have BFP. Up to date, various differential diagnoses have been reported for BFP, which are presented in Table 1.^15^ In our case, all these causes had been ruled out, thus a clinical diagnosis of bilateral Bell's palsy due to SARS‐CoV‐2 infection was made, and treatment was continued with corticosteroids, to which he responded successfully.

**TABLE 1:** Causes of bilateral facial palsy.

Infection	HIV, HSV, CMV, EBV, Rubella, Mumps, Varicella Zoster, Lyme disease, influenza, Syphilis
Trauma	Skull Fractures, parotid surgery
Metabolic	Diabetes mellitus
Neoplasm	Acute leukemia, neoplasm of the parotid gland, acoustic canal neoplasms, brainstem tumors
Autoimmune	Guillain–Barre syndrome, Sarcoidosis, Amyloidosis
Neurologic	Multiple Sclerosis, Parkinson's disease
Idiopathic	Bilateral Bell's palsy

According to a systematic search of available literature in PubMed, Scopus, and Embase databases using keywords including Bell's palsy, facial nerve palsy, COVID‐19, and SARS‐CoV‐2, we find four published case reports of isolated BFP (bilateral Bell's palsy) after COVID‐19.^7^, ^8^, ^9^, ^10^ Three of them are thought to be bilateral Bell's palsy post‐COVID‐19, the same as our case,^7^, ^8^, ^9^ and one is a case of delayed idiopathic BFP after COVID‐19 myocarditis.^10^ These cases and ours, go along with the definition of “probable association” of SARS‐CoV‐2 infection with neurologic complications, suggested by Ellu et al^16^ However, further longitudinal studies are needed to clarify the underlying mechanisms.

Although our patient had no sign of underlying diseases (as mentioned in Table 1), or any other neurologic disorders after COVID‐19, there are some case reports of BFP after COVID‐19 as a sign of GBS,^17^ or co‐infection with SARS‐CoV‐2 and EBV.^18^ Thus, in COVID‐19 patients with BFP, first of all, the mentioned differential diagnosis (Table 1) must be excluded.

In this case, we had a limitation. The best choice for imaging the peripheral nerves is MRI with contrast, which demonstrates increased enhancement in post‐contrast sequences in Bell's palsy (especially in the T1WI sequence).^19^ However, our patient refused to undergo it.

In conclusion, medical professionals should be aware of the possible neurologic complications in COVID‐19 patients and their top differential diagnosis.

## AUTHOR CONTRIBUTIONS

**Majid Dezfouli:** Conceptualization; project administration; supervision; writing – review and editing. **Firouzeh Moeinzadeh:** Project administration; supervision; writing – review and editing. **Farzin Ghiasi:** Project administration; supervision; writing – review and editing. **Neda Abdeyazdan:** Data curation; writing – original draft; writing – review and editing. **Sadegh Mazaheri‐Tehrani:** Conceptualization; data curation; investigation; writing – original draft; writing – review and editing.

## FUNDING INFORMATION

The authors would like to express that we have no funding received from any organization.

## CONFLICT OF INTEREST STATEMENT

The authors declare that they have no conflict of interest.

## ETHICAL APPROVAL

This study was approved by the institutional review board.

## CONSENT

Written informed consent was obtained from the patient for the publication of this case report.

## Data Availability

The data that support the findings of this study are available on request from the corresponding author.

## References

[1] WHO Coronavirus (COVID‐19) Dashboard . Accessed August 8, 2022. https://covid19.who.int

[2] Farshidfar F , Koleini N , Ardehali H . Cardiovascular complications of COVID‐19. JCI Insight. 2021;6(13):148980. doi:10.1172/jci.insight.148980 PMC8410051 34061779

[3] Rahman A , Niloofa R , Jayarajah U , De Mel S , Abeysuriya V , Seneviratne SL . Hematological abnormalities in COVID‐19: a narrative review. Am J Trop Med Hyg. 2021;104(4):1188‐1201. doi:10.4269/ajtmh.20-1536 33606667 PMC8045618

[4] Zacharias H , Dubey S , Koduri G , D'Cruz D . Rheumatological complications of Covid 19. Autoimmun Rev. 2021;20(9):102883. doi:10.1016/j.autrev.2021.102883 34237419 PMC8256657

[5] Mao L , Jin H , Wang M , et al. Neurologic manifestations of hospitalized patients with coronavirus disease 2019 in Wuhan, China. JAMA Neurol. 2020;77(6):683‐690. doi:10.1001/jamaneurol.2020.1127 32275288 PMC7149362

[6] Gupta S , Jawanda MK , Taneja N , Taneja T . A systematic review of Bell's palsy as the only major neurological manifestation in COVID‐19 patients. J Clin Neurosci. 2021;90:284‐292. doi:10.1016/j.jocn.2021.06.016 34275565

[7] Inui R , Fujiwara S , Kohara N , Kawamoto M . Post COVID‐19 bilateral facial nerve palsy. Intern Med. 2022;61(2):241‐243. doi:10.2169/internalmedicine.8448-21 34744110 PMC8851184

[8] Kerstens J , Deschuytere L , Schotsmans K , Maréchal E . Bilateral peripheral facial palsy following asymptomatic COVID‐19 infection: a case report. Acta Neurol Belg. 2021;121(3):815‐816. doi:10.1007/s13760-021-01665-7 33818743 PMC8020373

[9] Judge C , Moheb N , Castro Apolo R , Dupont JL , Gessner ML , Yacoub HA . Facial Diplegia as a rare late neurologic manifestation of SARS‐CoV‐2 infection. J Neurol Res. 2020;10(6):235‐236. doi:10.14740/jnr606 33984099 PMC8040456

[10] Matsumura K , Kawano H , Kurobe M , et al. Delayed acute Perimyocarditis and bilateral facial nerve palsy in a patient with COVID‐19. Intern Med. 2022;61(15):2327‐2332. doi:10.2169/internalmedicine.9752-22 35650137 PMC9424086

[11] Kim IS , Shin SH , Kim J , Lee WS , Lee HK . Correlation between MRI and operative findings in Bell's palsy and Ramsay hunt syndrome. Yonsei Med J. 2007;48(6):963‐968. doi:10.3349/ymj.2007.48.6.963 18159587 PMC2628199

[12] Dalaqua M , do Nascimento FBP , Miura LK , MRT G , Barbosa Junior AA , Reis F . Magnetic resonance imaging of the cranial nerves in infectious, neoplastic, and demyelinating diseases, as well as other inflammatory diseases: a pictorial essay. Radiol Bras. 2022;55(1):38‐46. doi:10.1590/0100-3984.2021.0042 35210663 PMC8864690

[13] Islamoglu Y , Celik B , Kiris M . Facial paralysis as the only symptom of COVID‐19: a prospective study. Am J Otolaryngol. 2021;42(4):102956. doi:10.1016/j.amjoto.2021.102956 33592554 PMC7972325

[14] Eviston TJ , Croxson GR , Kennedy PG , Hadlock T , Krishnan AV . Bell's palsy: aetiology, clinical features and multidisciplinary care. J Neurol Neurosurg Psychiatry. 2015;86(12):1356‐1361. doi:10.1136/jnnp-2014-309563 25857657

[15] Jung J , Park DC , Jung SY , Park MJ , Kim SH , Yeo SG . Bilateral facial palsy. Acta Otolaryngol. 2019;139(10):934‐938. doi:10.1080/00016489.2019.1651134 31430217

[16] Ellul MA , Benjamin L , Singh B , et al. Neurological associations of COVID‐19. Lancet Neurol. 2020;19(9):767‐783. doi:10.1016/S1474-4422(20)30221-0 32622375 PMC7332267

[17] Szewczyk AK , Skrobas U , Jamroz‐Wiśniewska A , Mitosek‐Szewczyk K , Rejdak K . Facial Diplegia‐complication or manifestation of SARS‐CoV‐2 infection? A case report and systemic literature review. Healthcare (Basel). 2021;9(11):1492. doi:10.3390/healthcare9111492 34828542 PMC8618007

[18] Cabrera Muras A , Carmona‐Abellán MM , Collía Fernández A , Uterga Valiente JM , Antón Méndez L , García‐Moncó JC . Bilateral facial nerve palsy associated with COVID‐19 and Epstein‐Barr virus co‐infection. Eur J Neurol. 2021;28(1):358‐360. doi:10.1111/ene.14561 32997868 PMC7537085

[19] Romano N , Federici M , Castaldi A . Imaging of cranial nerves: a pictorial overview. Insights. Imaging. 2019;10(1):33. Published 2019 Mar 15. doi:10.1186/s13244-019-0719-5 PMC6420596 30877408

